# A Genomic Signature Reflecting Fibroblast Infiltration Into Gastric Cancer Is Associated With Prognosis and Treatment Outcomes of Immune Checkpoint Inhibitors

**DOI:** 10.3389/fcell.2022.862294

**Published:** 2022-04-26

**Authors:** Yi Lu, Dan Li, Yixin Cao, Leqian Ying, Qing Tao, Fen Xiong, Zhangmin Hu, Yufei Yang, Xuehan Qiao, Chen Peng, Dongqin Zhu, Deqiang Wang, Xiaoqin Li

**Affiliations:** ^1^ Department of Oncology, The Affiliated Hospital of Jiangsu University, Zhenjiang, China; ^2^ Department of Hematology, Shaoxing Hospital, Zhejiang University School of Medicine, Zhejiang, China; ^3^ Nanjing Geneseeq Technology Inc., Nanjing, China

**Keywords:** fibroblasts, immunotherapy, mutation, tumor microenvironment, gastric cancer

## Abstract

**Background:** The immunotherapy efficacy in gastric cancer (GC) is limited. Cancer-associated fibroblasts (CAFs) induce primary resistance to immunotherapy. However, CAF infiltration in tumors is difficult to evaluate due to the lack of validated and standardized quantified methods. This study aimed to investigate the impact of infiltrating CAFs alternatively using fibroblast-associated mutation scoring (FAMscore).

**Methods:** In a GC cohort from Affiliated Hospital of Jiangsu University (AHJU), whole exon sequencing of genomic mutations, whole transcriptome sequencing of mRNA expression profiles, and immunofluorescence staining of tumor-infiltrating immune cells were performed. GC data from The Cancer Genome Atlas were used to identify genetic mutations which were associated with overall survival (OS) and impacted infiltrating CAF abundance determined by transcriptome-based estimation. FAMscore was then constructed through a least absolute shrinkage and selection operator Cox regression model and further validated in AHJU. The predictive role of FAMscore for immunotherapy outcomes was tested in 1 GC, one melanoma, and two non-small-cell lung cancer (NSCLC-1 and -2) cohorts wherein participants were treated by immune checkpoint inhibitors.

**Results:** FAMscore was calculated based on a mutation signature consisting of 16 genes. In both TCGA and AHJU, a high FAMscore was an independent predictor for poor OS of GC patients. FAMscore was associated with immune-associated genome biomarkers, immune cell infiltration, and signaling pathways of abnormal immunity. Importantly, patients with high FAMscore presented inferiority in the objective response rate of immunotherapy compared to those with low *FAMscore*, with 14.6% vs. 66.7% (p<0.001) in GC, 19.6% vs. 68.2% (p<0.001) in NSCLC-1, 23.1% vs 75% (p = 0.007) in NSCLC-2, and 40.9% vs 75% (p = 0.037) in melanoma. For available survival data, a high FAMscore was also an independent predictor of poor progression-free survival in NSCLC-1 (HR = 2.55, 95% CI: 1.16–5.62, *p* = 0.02) and NSCLC-2 (HR = 5.0, 95% CI: 1.13–22.19, *p* = 0.034) and poor OS in melanoma (HR = 3.48, 95% CI: 1.27–9.55, *p* = 0.015).

**Conclusions:** Alternative evaluation of CAF infiltration in GC by determining the FAMscore could independently predict prognosis and immunotherapy outcomes. The FAMscore may be used to optimize patient selection for immunotherapy.

## Introduction

According to the Global Cancer Statistics, gastric cancer (GC) was diagnosed in approximately 1,034,000 individuals and led to 768,793 deaths worldwide in 2020. GC is the fifth most common cancer after breast, lung, colorectal, and prostate cancers and is the fourth leading cause of cancer-related deaths (Sung et al., 2021). Although the incidence of GC has declined over the past decades and has reached a plateau, however, it still poses a considerable health threat globally despite numerous prophylactic and therapeutic improvements.

GC is phenotypically and genotypically heterogeneous (Cancer Genome Atlas Research Network, 2014; Cristescu et al., 2015) and possesses a complex tumor microenvironment (TME) of tumor cells, cancer-associated fibroblasts (CAFs), tumor-associated macrophages, other infiltrating immune cells, endothelial cells, extracellular matrix proteins, and signaling molecules, such as cytokines and chemokines. Many components of the TME play critical roles in tumor development and progression. Of these, CAFs structurally and functionally affect tumor initiation, tumor cell proliferation, angiogenesis, extracellular matrix remodeling, and metastasis through a variety of signaling pathways (Ham et al., 2019; Melissari et al., 2020).

In TME, the anti-cancer immune response is mediated primarily by CD8+ T cells, which are controlled by co-inhibitory signaling molecules, including programmed cell death protein 1 ligand 1 (PD-L1) and cytotoxic T lymphocyte-associated protein 4 (CTLA-4). These two checkpoint molecules have served as targets for immunotherapy. However, immune checkpoint inhibitors (ICIs) targeting the co-inhibitory signaling pathways in T cells and tumor cells have helped in only a minority of patients with GC (Smyth et al., 2021).

Recently, growing evidence has shown that CAFs can modulate immune cell activity and suppress the anti-cancer immune response. For example, CAFs promote the recruitment and expansion of pro-tumoral immune cell populations, as shown by an increased Th2 or CD4^+^ Th17 T cell response (Su et al., 2010; Shani et al., 2020). CAFs exert immunosuppressive activity by inhibiting the proliferation of effector T lymphocytes and reducing CD8+ T-cell infiltration within the tumor islets (Feig et al., 2013; Cremasco et al., 2018). CAFs also impact the expression of immune checkpoint ligands, such as PD-1, PD-L1, CTLA-4, T-cell immunoglobulin, and mucin domain-containing protein-3 (TIM3) (Kieffer et al., 2020). Based on the knowledge of CAF biology, CAFs have been further revealed to be associated with primary resistance of patients to immunotherapy in several cancers (Kieffer et al., 2020; Galbo et al., 2021; Mhaidly and Mechta-Grigoriou, 2021).

In GC, CAF infiltration is associated with diffused and genomic stable subtypes and has a deteriorated survival impact (Liu et al., 2021). CAFs may induce immunosuppressive TME potentially by activating TGF-β signaling (Liu et al., 2021), the main driver of immune exclusion. In GC xenograft models, targeting CAFs substantially enhanced the antitumor effects of ICIs (Wen et al., 2017). However, the impact of CAF infiltration levels on ICI efficacy in GC patients remains unclear.

Some factors limit the wide investigation of the compromised efficacy of immunotherapy caused by CAF infiltration. First, tumor tissues are sometimes unavailable for recurrent or metastatic diseases. Meanwhile, samples from puncture biopsies are usually too small to fulfill a request for CAF detection. Second, the present primary method for detecting CAFs, immunohistochemistry (IHC), still needs to be standardized and validated. In particular, the optimal cutoff to divide high and low abundance of infiltrating CAFs in tumors is still unclear. Third, another accepted method to evaluate CAF abundance, computational algorithms based on transcriptome (Aran et al., 2017), is costly and vulnerable to RNA-seq quality. Moreover, intratumoral heterogeneity can result in different detection results.

It is necessary to develop an efficient, cost-effective, and well-established tool to evaluate the impact of CAF infiltration in order to promote CAF-associated studies and clinical practice. At present, the detection of genetic mutations by PCR or next-generation sequencing (NGS) has been regularly adopted to improve clinical decisions. Mutations in specific genes have been linked to the immune phenotype. For example, TP53 mutations in patients with myelodysplastic syndromes and secondary acute myeloid leukemia were significantly associated with reduced numbers of cytotoxic T cells, helper T cells, and natural killer (NK) cells but increased immunosuppressive regulatory T cells (Tregs) in the immune microenvironment (Sallman et al., 2020). Thus, we wondered whether CAF abundance in tumors was impacted and therefore could be reflected by genomic mutations, which remains unknown.

In this study, fibroblast-associated mutations (FAMs) in GC were identified, and those with the most prominent prognostic role were used to construct a genetic signature. Scoring based on this signature, named FAMscore, was shown to reflect TME well and could be used to predict immunotherapy outcomes.

## Patients and Methods

### GC Cohorts for Construction of the FAM Signature

A total of two GC cohorts with whole-exome sequencing (WES) data were used to construct the FAM signature, the one from The Cancer Genome Atlas (TCGA) as the discovery set, and the other from Affiliated Hospital of Jiangsu University (AHJU) as the validation set. The enrollment criteria for patients included the following: 1) matched transcriptome and WES data for FAM signature construction in the TCGA discovery set; 2) available WES data for FAMscore calculation; 3) pathological diagnosis of GC; and 4) no prior history of anti-cancer therapies (including neoadjuvant therapy). Clinical and clinicopathological classifications and staging were based on the American Joint Committee on Cancer criteria. The research protocol of the AHJU cohort was approved by the Ethics Committee of AHJU, and all patients provided written informed consent.

### Multiomic Data Acquirement and Storage

Multidimensional data of TCGA, including mRNA expression profiles, somatic mutations, and microsatellite instability (MSI), were downloaded from the cBioPortal database (http://www.cbioportal.org/). Tumor neoantigen burden (TNB) was obtained from the immune landscape of TCGA pan-cancer (Thorsson et al., 2018). Transcriptome data of AHJU were stored in the dataset-EGAD00001004164 in the European Genome-phenome Archive. Genome data of AHJU were stored in the dataset-HRA001647 in the Genome Sequence Archive for Human.

### Immunotherapy Cohorts

A total of four published immunotherapy cohorts of solid tumors with available data on WES and response evaluation and/or survival were downloaded and analyzed to determine the role of FAMscore, including 1 GC cohort wherein the participants had received PD-1 inhibition with pembrolizumab (Kim et al., 2018), one melanoma cohort wherein participants were treated with pembrolizumab or nivolumab (Hugo et al., 2017), one non-small-cell lung cancer (NSCLC) cohort (NSCLC-1) wherein participants were treated with combined PD-1 (nivolumab) and CTLA-4 (ipilimumab) blockade (Hellmann et al., 2018), and another NSCLC cohort (NSCLC-2) wherein participants were treated with pembrolizumab alone (Rizvi et al., 2015).

### RNA-Seq

A total of 34 patients with GC underwent RNA-seq in the AHJU cohort. The RNA-seq process has been reported previously (Duan et al., 2020). In brief, the library was prepared using the KAPA Stranded RNA-seq Kit with RiboErase (KAPA Biosystems, United States), after extraction of total RNA from fresh samples and depletion of ribosomal RNA. Then, the library was sequenced on Illumina HiSeq4000 NGS platforms (Illumina, United States), after determination of the library concentration and assessment of library quality. The sequence reads were generated by base calling performed on bcl2fastq v2.16.0.10 (Illumina, United States) in the FASTQ format (Illumina 1.8 + encoding). Following quality control and isoform and gene-level quantification, the transcriptome was mapped using STAR (version 2.5.3a).

### WES

A total of 74 patients with GC underwent WES in the AHJU cohort. As previously described (Wang et al., 2021), after genomic DNA extraction, the whole genome library was prepared using the KAPA Hyper Prep Kit (KAPA Biosystems), and exome capture was performed using the Illumina Rapid Capture Extended Exome Kit (Illumina Inc.). Then, enriched libraries were sequenced on the Illumina HiSeq4000 NGS platform (Illumina, United States) as paired 150-bp reads. The data processing has been described elsewhere (Xu et al., 2018).

### Transcriptome-Based Immune Infiltration Estimation

We selected the xCell algorithm to quantify the abundance of infiltrating cells in the TME based on transcriptome data. It uses a novel technique for reducing associations between closely related cell types and validates signatures using both *in silico* simulations and cytometry immunophenotyping (Aran et al., 2017).

### Selection of Genomic Variants

We included somatic nonsynonymous variants (SNVs) in coding regions for analysis, including frameshift, missense, nonsense, and splice-site mutations, which may be functional (Devarakonda et al., 2018). The tumor mutation burden (TMB) was calculated as the number of SNVs.

### FAMscore Construction

To facilitate detection, genes were asked to have a mutation frequency of ≥5%. Mutations (no matter the number) that occurred in one gene of the same sample were evaluated as 1 or 0. Genetic mutations associated with overall survival (OS) were selected using univariate Cox regression models. Then, CAF abundances between the genetically mutated type and wild-type were compared, and genes with mutations that significantly impacted CAF abundance were selected. Furthermore, genes with the most useful prognostic mutations were screened using the least absolute shrinkage and selection operator (LASSO) Cox regression model, and a FAMscore model was subsequently built based on the fraction of selected genes using Cox regression coefficients. The formula is established as follows: FAMscore = sum (each gene’s mutation × corresponding coefficient).

### MSI Detection

After genomic DNA extraction from both GC and normal tissues, the MSI status was determined by single fluorescent multiplex PCR based on five well-known mononucleotide repeats, BAT-25, BAT-26, NR-21, NR-24, and NR-27 (Cristescu et al., 2015). MSI was defined as allelic size variations in at least two microsatellites in samples or microsatellite stability (MSS).

### Gene Set Enrichment Analysis (GSEA)

Based on the criteria of adjusted *p*-value<0.05 and log2 (fold change) > 1, differentially expressed genes (DEGs) were determined between the high and low FAMscore subgroups. NetworkAnalyst 3.0 (https://www.networkanalyst.ca/) was used for GSEA based on the Kyoto Encyclopedia of Genes and Genomes (KEGG).

### Multiple-Immunofluorescence (mIF) Staining

Antibodies such as anti-CD8 (CST70306, Cell Signaling Technology, United States), anti-CD56 (CST3576), anti-panCK (CST4545), anti-S100 (ab52642, Abcam, UK), anti-HLA-DR (ab92511), and anti-CD68 (BX50031, Biolynx, China) were used in mIF using the PANO 7-plex IHC kit (Panovue, Beijing, China), according to the manufacturer’s instructions. The Mantra System (PerkinElmer, Waltham, MA, United States) was used to scan the stained slides and reconstruct the images of the sections. inForm image analysis software (PerkinElmer, Waltham, MA, United States) was used to quantify the cells in the images.

### Statistical Analysis

The χ^2^ test, Student’s *t*-test, Fisher’s exact probability test, and Mann–Whitney *U* test were used for comparisons between groups. The Pearson correlation coefficient R was used to evaluate the correlations between groups. The Kaplan–Meier method and log-rank test were used for survival analysis. The optimal cut-off values to define high and low FAMscore in TCGA and AHJU cohorts were determined based on the association of FAMscore with OS using the *survminer* R package. Univariate and multivariate Cox proportional hazard models were used to evaluate prognostic factors, and hazard ratios (HRs) along with their 95% confidence intervals (CIs) were determined. The receiver operating characteristic curve (ROC) and the areas under the ROC curves (AUCs) were used to evaluate the predictive power of FAMscore for the objective response of patients to immunotherapy. Statistical significance was set at *p* < 0.05. R (version 4.0.1), R Bioconductor packages, and SPSS (version 19.0, Chicago, IL) were used in the analyses.

## Results

### Patient Characteristics

In the study, we included 383 patients from TCGA and 74 patients from AHJU. An overview of the patient characteristics is provided in Table 1, and the details are in Supplementary Table S1. No significant differences were found in patient characteristics between the TCGA and AHJU cohorts.

**TABLE 1:** Patient characteristics.

Characteristic	AHJU	TCGA^*^	*p*-value
Age			
<65 years	28 (37.8)	164 (43.2)	0.397
≥65 years	46 (62.2)	216 (56.8)	
Gender			
Female	19 (25.7)	133 (34.7)	0.130
Male	55 (74.3)	250 (65.3)	
Stage			
I/II	28 (37.8)	175 (46.4)	0.175
III/IV	46 (62.2)	202 (53.6)	
Histology grade			
I/II	21 (28.4)	146 (39.0)	0.083
III	53 (71.6)	228 (61.0)	
MSI status			
MSS	65 (87.8)	321 (83.8)	0.381
MSI	9 (12.2)	62 (16.2)	

AHJU, Affiliated Hospital of Jiangsu University; TCGA, The Cancer Genome Atlas.

MSI: microsatellite instability; MSS: microsatellite stability.

^*^Based on available data.

### Derivation of the FAMscore and Its Association With Patient Characteristics

**FIGURE 1:**
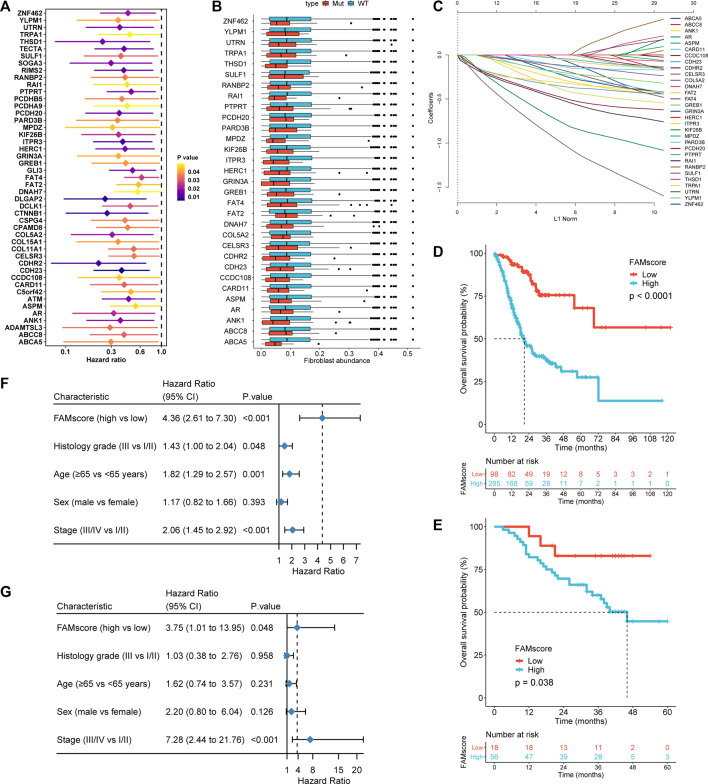
FAMscore construction and its prognostic role. **(A)** Hazard ratios of genes with mutations in univariate Cox models that were significantly associated with overall survival. **(B)** Genes whose mutation and wild-type subgroups had significantly different CAF abundance into tumors (all *p* < 0.05). **(C)** LASSO coefficient profiles of the fractions of genes in B. **(D)** and **(E)** High FAMscore was significantly associated with poor overall survival (OS) in TCGA **(D)** and AHJU **(E-G)** High FAMscore was an independent risk factor for OS in multivariate Cox regression models in TCGA **(F)** and AHJU **(G)**. Grade: histology grade; CAF: cancer-associated fibroblast; LASSO: least absolute shrinkage and selection operator; AHJU: Affiliated Hospital of Jiangsu University; TCGA: The Cancer Genome Atlas.

In the TCGA GC cohort, 828 genes presented a mutation frequency of ≥5% (Supplementary Table S2). Of these, mutations in 47 genes were significantly associated with OS (Figure 1A), and the CAF abundances calculated by the xCell algorithm (Supplementary Table S3) were significantly different between the mutated type and wild-type in 31 of these genes (Figure 1B). After LASSO Cox regression analysis (Figure 1C), 16 genes were selected to construct the FAMscore of OS, as follows: FAMscore = ANK1 mutation*-0.211 + AR mutation*-0.612 + CARD11 mutation*-0.077 + CDH23 mutation*-0.313 + CDHR2 mutation*-0.421 + COL5A2 mutation*-0.088 + FAT2 mutation*-0.033 + GRIN3A mutation*-0.134 + ITPR3 mutation*-0.287 + PARD3B mutation*-0.154 + PCDH20 mutation*-0.402 + PTPRT mutation*-0.019 + THSD1 mutation*-0.803 + UTRN mutation*-0.288 + YLPM1 mutation*-0.104 + ZNF462 mutation*-0.151.

The FAMscore in TCGA and AHJU is provided in Supplementary Table S1. In TCGA, FAMscore was significantly higher in histological grade III than in grade I/II (*p* = 0.047; Supplementary Figure S1A). A similar result was observed in AHJU, but the significance was limited by the sample size (*p* = 0.22; Supplementary Figure S1B). The FAMscore was not significantly associated with the other patient characteristics including age, sex, and stage.

### FAMscore Is an Independent Prognostic Factor

In TCGA, high FAMscore was significantly associated with poor OS (*p* < 0.0001; Figure 1D), which was further validated in AHJU (*p* = 0.038; Figure 1E). In multivariate analysis, high FAMscore was an independent predictor of poor OS in both TCGA (HR = 4.36, 95% CI: 2.61–7.30, *p* < 0.001; Figure 1F) and AHJU (HR = 3.75, 95% CI: 1.01–13.95, *p* = 0.048; Figure 1G).

### FAMscore Is Associated With Immune Indexes

**FIGURE 2:**
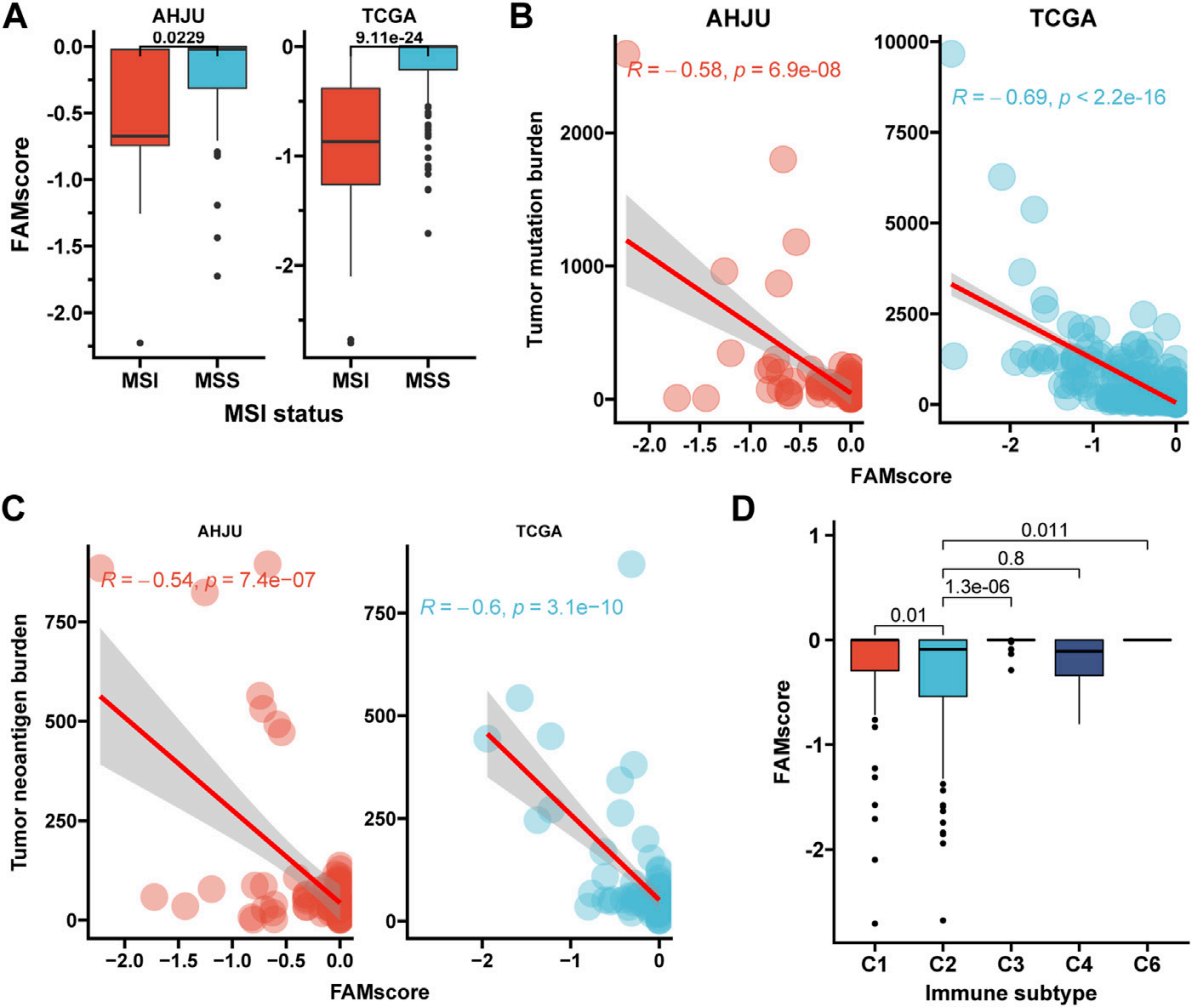
FAMscore and immune-associated indexes. **(A)** FAMscore by the MSI status; **(B,C)** correlations of FAMscore with tumor mutation **(B)** and neoantigen burdens **(C)**; **(D)** FAMscore by the immune subtype. MSI: microsatellite instability.

MSI GC had a significantly lower FAMscore than MSS GC in both TCGA (*p* = 9.11e-24) and AHJU (*p* = 0.023) (Figure 2A). TMB (Figure 2B) and TNB (Figure 2C) were negatively correlated with FAMscore in both TCGA (R = -0.69, *p* < 2.2e-16 and R = -0.6, *p* = 3.1e-10, respectively) and AHJU (R = -0.58, *p* = 6.9e-08 and R = -0.54, *p* = 7.4e-07, respectively). Moreover, the immune subtype C2 (IFN-gama dominant) in TCGA, which may override an evolving type I immune response (Thorsson et al., 2018), had the lowest FAMscore among immune subtypes (Figure 2D).

### Transcriptome Features Associated With FAMscore

**FIGURE 3:**
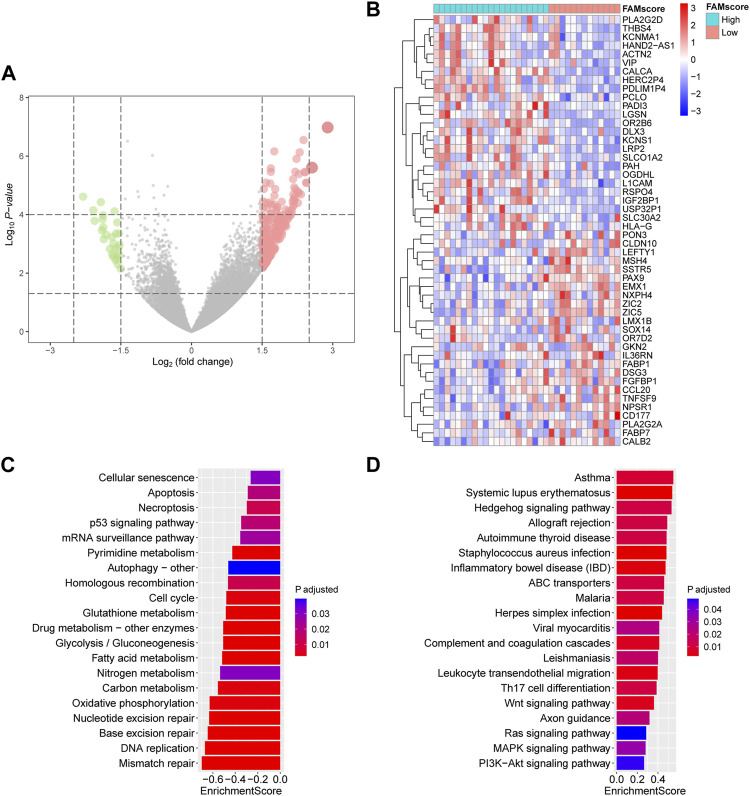
Transcriptome features associated with FAMscore. **(A)** Volcano plot for differentially expressed genes. **(B)** Heatmap for top 50 differentially expressed genes between low and high FAMscore subgroups. **(C,D)** Selected KEGG pathways in GSEA of low **(C)** and high **(D)** FAMscore subgroups. KEGG: Kyoto Encyclopedia of Genes and Genomes; GSEA: gene set enrichment analysis.

A total of 180 DEGs were identified between the high and low FAMscore subgroups in the AHJU cohort (Figure 3A and Supplementary Table S4), and the top 50 DEGs are shown in Figure 3B. GSEA showed that genes in KEGG pathways involving DNA repair, cell death, cell cycle, and aerobic metabolism were significantly enriched in the low FAMscore subgroup (Figure 3C), while those involving autoimmune diseases, infection, and signaling associated with malignant biology were significantly enriched in the high FAMscore subgroup (Figure 3D). These results suggest that GC with a high FAMscore is more aggressive and characterized by abnormal immunity.

### FAMscore Is Associated With Immune Infiltration

**FIGURE 4:**
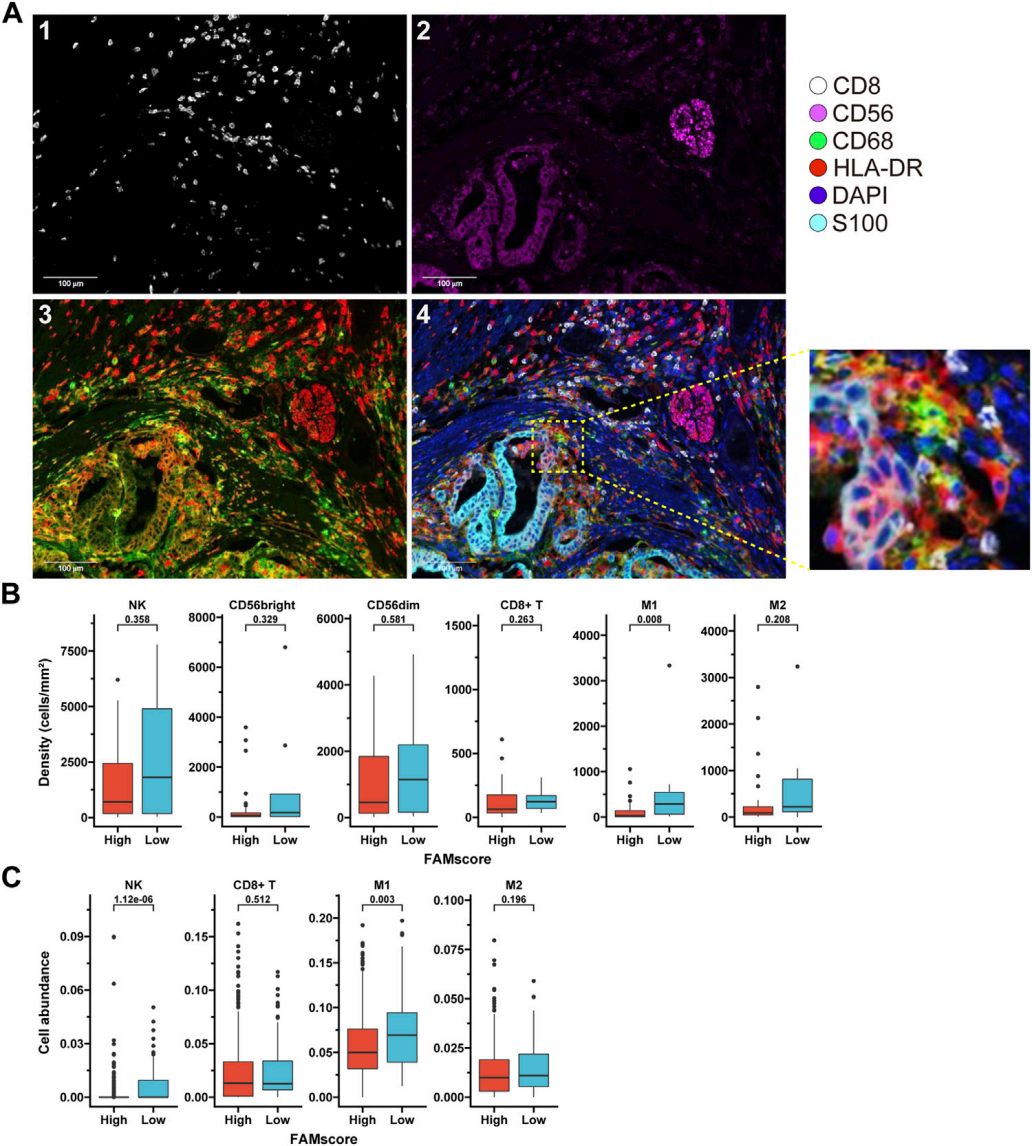
FAMscore and immune infiltration. **(A)** mIF staining of biomarkers on the surface of immune cells infiltrated into tumors. 1: CD8 staining; 2: CD56 staining; 3: HLA-DR (red) and CD68 (green) staining; and 4: reconstructed image including all biomarkers. **(B)** Density of infiltrating immune cells in tumor parenchyma from the AHJU cohort, stratified by the FAMscore level. **(C)** Abundance of infiltrating immune cells in gastric cancer tissues from TCGA calculated by xCell based on transcriptome, stratified by the FAMscore level. mIF: multiple immunofluorescence; AHJU: Affiliated Hospital of Jiangsu University; TCGA: The Cancer Genome Atlas.

mIF for selected immune cells was performed in samples from 47 patients in the AHJU cohort (Figure 4A and Supplementary Table S5). We found that the density of M1 macrophages in the tumor parenchyma was significantly higher in the low FAMscore subgroup than that in the high FAMscore subgroup (*p* = 0.008; Figure 4B). The low FAMscore subgroup also had a nonsignificantly increased density of NK cells (including CD56bright and CD56dim subtypes) in the tumor parenchyma. In TCGA (Figure 4C and Supplementary Table S6), cell abundances calculated by the xCell algorithm further verified that low FAMscore was significantly associated with increased infiltration of M1 macrophages (*p* = 0.003) and NK cells (*p* = 1.12e-06). These results indicate that a low FAMscore may reflect a favorable TME characterized by dynamic immune infiltration.

### FAMscore Is Associated With Responses to Immunotherapy

**FIGURE 5:**
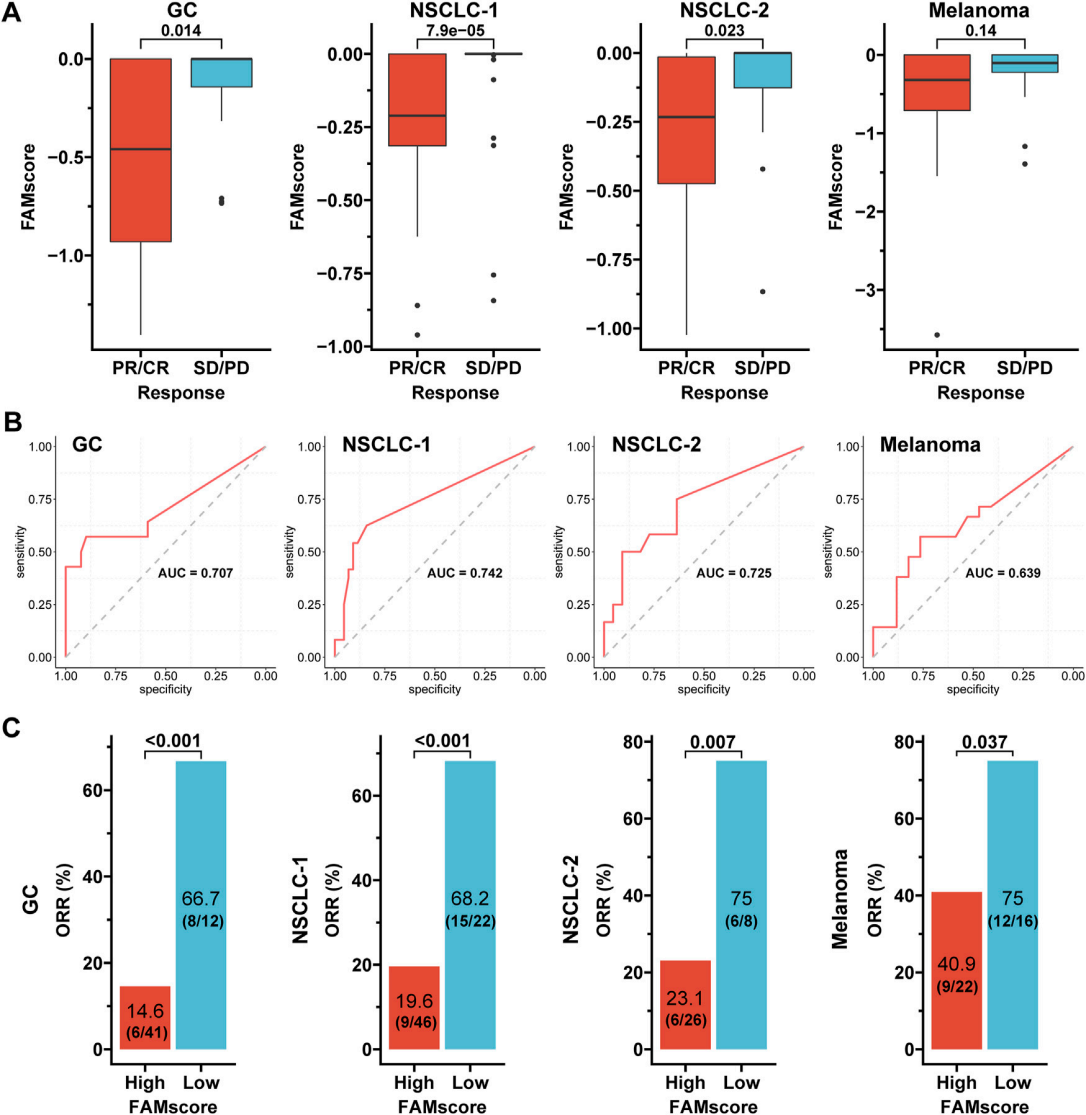
FAMscore and immunotherapy response in four cohorts. **(A)** FAMscore stratified by the response status. **(B)** ROC curves evaluated the predictive ability of FAMscore for immunotherapy response. **(C)** Immunotherapy responses stratified by the FAMscore level. GC: gastric cancer; NSCLC: non-small-cell lung cancer; PR: partial response; CR: complete response; SD: stable disease; PD: progressive disease; ROC: receiver operating characteristics; AUC: area under the curve; ORR: objective response rate.

In four immunotherapy cohorts (Supplementary Table S7), patients were divided into partial response/complete response (PR/CR) and stable disease/progressive disease (SD/PD) subgroups. The PR/CR subgroups generally had lower FAMscores than the SD/PD subgroups (*p* = 0.014 for the GC cohort, *p* = 7.9e-05 for the NSCLC-1 cohort, *p* = 0.023 for the NSCLC-2 cohort, and *p* = 0.14 for the melanoma cohort; Figure 5A). The ROC analysis showed that the AUC for the prediction of FAMscore to immunotherapy responses ranged from 0.639 to 0.742 (Figure 5B). Based on the optimal threshold of FAMscore for the maximum ROC curve values (−0.315, −0.002, −0.3, and −0.225 for the GC, NSCLC-1, NSCLC-2, and melanoma cohorts, respectively), patients were dichotomized into high and low FAMscore subsets. Furthermore, the objective response rates (ORRs) between the low and high FAMscore subgroups were 66.7 vs. 14.6% (8/12 vs. 6/41, *p* < 0.001) for the GC cohort, 68.2 vs. 19.6% (15/22 vs. 9/46, *p* < 0.001) for the NSCLC-1 cohort, 75 vs. 23.1% (6/8 vs. 6/26, *p* = 0.007) for the NSCLC-2 cohort, and 75 vs. 40.9% (12/16 vs. 9/22, *p* = 0.037) for the melanoma cohort (Figure 5C). These findings clearly indicate that the FAMscore was correlated with immunotherapy responses.

### FAMscore Is Associated With Prognosis of Immunotherapy

**FIGURE 6:**
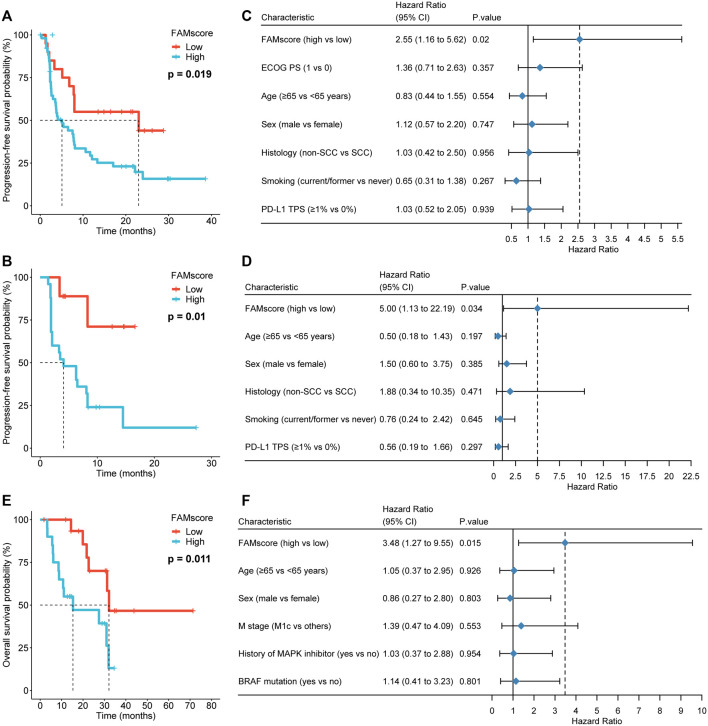
FAMscore and prognosis in immunotherapy. **(A–B)** Association of FAMscore with progression-free survival (PFS) in Kaplan–Meier survival analyses in the NSCLC-1 **(A)** and NSCLC-2 **(B)** cohorts. **(C–D)** Association of FAM score with PFS in multivariate Cox models in the NSCLC-1 **(C)** and NSCLC-2 **(D)** cohorts. **(E–F)** Association of FAMscore with overall survival in the Kaplan–Meier survival analysis **(E)** and multivariate Cox model of the melanoma cohort **(F)**. NSCLC: non-small-cell lung cancer; ECOG PS: Eastern Cooperative Oncology Group performance status; TPS: tumor proportion score.

Progression-free survival (PFS) was available in the NSCLC-1 and NSCLC-2 cohorts, and OS was available for the melanoma cohort. A high FAMscore was associated with poor PFS in both the NSCLC-1 (*p* = 0.019) and NSCLC-2 (*p* = 0.01) cohorts (Figure 6A,B). In multivariate analysis including PD-L1 expression, smoking history, and other clinical features, high FAMscore was the only independent predictor of poor PFS in both the NSCLC-1 (HR = 2.55, 95% CI: 1.16–5.62, *p* = 0.02; Figure 6C) and NSCLC-2 (HR = 5.0, 95% CI: 1.13–22.19, *p* = 0.034; Figure 6D) cohorts. Moreover, a high FAMscore was also associated with poor OS of immunotherapy in the melanoma cohort (*p* = 0.011; Figure 6E). In multivariate analysis including BRAF mutation, therapy history of MAPK inhibitors, and other clinical features, a high FAMscore was still the only independent predictor of poor OS (HR = 3.48, 95% CI: 1.27–9.55, *p* = 0.015; Figure 6F). These results revealed that the FAMscore was a strong predictor of immunotherapy-associated survival.

## Discussion

CAFs induce primary resistance to immunotherapy (Kieffer et al., 2020; Galbo et al., 2021; Mhaidly and Mechta-Grigoriou, 2021), while the evaluation of CAFs in tumors is still considered difficult and lacks standard and validated quantified methods. In this study, a genomic mutation signature associated with CAF infiltration was defined, and a novel method, named FAMscore, was constructed to evaluate CAF infiltration. We showed that FAMscore was a robust biomarker for predicting prognosis in GC and immunotherapy outcomes in several tumor types. FAMscore may be used to guide more effective immunotherapy strategies in solid tumors, including GC.

We revealed that a high FAMscore was an independent predictor of poor OS, probably reflecting the role of CAFs in GC invasion and metastasis, the master of prognosis. CAFs in GC have been found to secrete cytokines, such as CXCL12, interleukin 11 (IL-11), stromal-derived factor 1 (SDR1), and fibroblast growth factor 9 (FGF-9), to promote epithelial–mesenchymal transition (EMT), the key initiator of tumor invasion and metastasis (Ham et al., 2019). Interestingly, CAFs may also be a major source of angiogenic factors that contribute to GC angiogenesis, and CAF-derived IL-6, IL-8, and IL-11 mediate resistance to chemotherapy (Ham et al., 2019; Zhai et al., 2019). The association of FAMscore with the efficacy of angiogenesis inhibitors or chemotherapy needs to be investigated.

Previously, CAFs were reported to promote M2 polarization of macrophages and cause the accumulation of M2 macrophages into tumors (Zhang et al., 2019). In our study, a high FAMscore was associated with a decreased infiltration of M1 macrophages compared to a low FAMscore, which may reflect the reduced M1 polarization induced by CAFs. Moreover, the NK-cell phenotype and antitumor cytotoxicity were modulated by CAFs (Balsamo et al., 2009; Zhang et al., 2019). Our study showed that a high FAMscore was also associated with decreased infiltration of NK cells, indicating that CAFs may impede NK-cell infiltration in GC. Interestingly, FAMscore was not associated with the infiltration of CD8^+^ T cells. Recent studies also found that the infiltration of CD8^+^ T cells was not associated with TMB (Jiang et al., 2019), and NK cell rather than CD8^+^ T cell infiltration reflected favorable TME (Duan et al., 2020). The potential explanation may be relevant to T-cell dysfunction.

Recently, TMB has been proposed as a predictive biomarker for response to ICIs (Chan et al., 2019) because genetic mutations may generate immunogenic neoantigens (Gubin et al., 2015). Based on the phase 2 KEYNOTE-158 study (Marabelle et al., 2020), the U.S. Food and Drug Administration (FDA) approved pembrolizumab for the treatment of patients with high TMB across nine cancer types. However, a recent study failed to validate the predictive role of TMB in several cancer types and revealed that high TMB showed predictive accuracy for ICI response only in cancer types whose CD8^+^ T-cell infiltration was positively correlated with neoantigen load (McGrail et al., 2021). These findings indicate that only mutations impacting immune infiltration are effective and can be considered biomarkers of immunotherapy. This can be verified by FAMscore in our study, an alternative evaluation of CAF infiltration by genetic mutations, simultaneously reflecting NK-cell and macrophage M1 infiltration, which presented strong associations with both response and survival of ICI treatments.

Our FAMscore was calculated based on a signature of 16 genes. Of them, AR has been demonstrated to negatively regulate PD-L1 expression, and therefore lower AR-expressed tumors achieved a better response to immunotherapy in hepatocellular carcinoma (Jiang et al., 2020). In addition, patients with PTPRT mutations harbored favorable ORR and survival outcomes in several tumor types (Zhang et al., 2022). However, the associations of other genes used in our model with ICI efficacy have not been reported, although some of them have been found to play critical roles in cancer, such as COL5A2 (Han et al., 2022), FAT2 (Gao et al., 2014), ITPR3 (Wu et al., 2021), PCDH20 (Wang et al., 2016), and UTRN (Zhou et al., 2021). Moreover, the potential mechanisms underlying the impact of these genes on CAF infiltration remain unknown, which needs to be investigated.

This study has several limitations. First, FAMscore was constructed based on GC data in this study, and other tumor types may require proprietary FAMscore considering tumor heterogeneity, although we showed a consistent predictive role of FAMscore for immunotherapy outcomes among the three tumor types. Second, it is still unclear why mutations in our FAM signature can impact CAF infiltration; further experimental research is needed. Moreover, FAMscore was calculated based on genetic mutations whose detection can be impacted by sample quality, sequencing platforms, mutation-calling algorithms, and other confounding factors.

In conclusion, we developed a convenient method to evaluate the impact of CAF infiltration on clinical outcomes based on well-established genetic mutation detection techniques. Current evidence supports the potential use of the FAMscore as a biomarker for ICI treatment in GC. The FAM signature should be further explored in other tumor types to build tumor type-specific FAMscores in order to optimize patient selection of immunotherapy more widely.

## Data Availability

The datasets presented in this study can be found in online repositories. The names of the repository/repositories and accession number(s) can be found in the article/Supplementary Material.

## Supplementary Material

Supplementary Figure S1 FAMscore and clinical features. **A** and **B**: FAMscore by clinical features in TCGA (D) and AHJU (E). AHJU: Affiliated Hospital of Jiangsu University; TCGA: The Cancer Genome Atlas.
