# Supplementary figures and images for: A Genomic Signature Reflecting Fibroblast Infiltration Into Gastric Cancer Is Associated With Prognosis and Treatment Outcomes of Immune Checkpoint Inhibitors
Source: Front Cell Dev Biol. 2022 Apr 26;10:862294. doi: 10.3389/fcell.2022.862294 (PMC9087633; doi:10.3389/fcell.2022.862294)

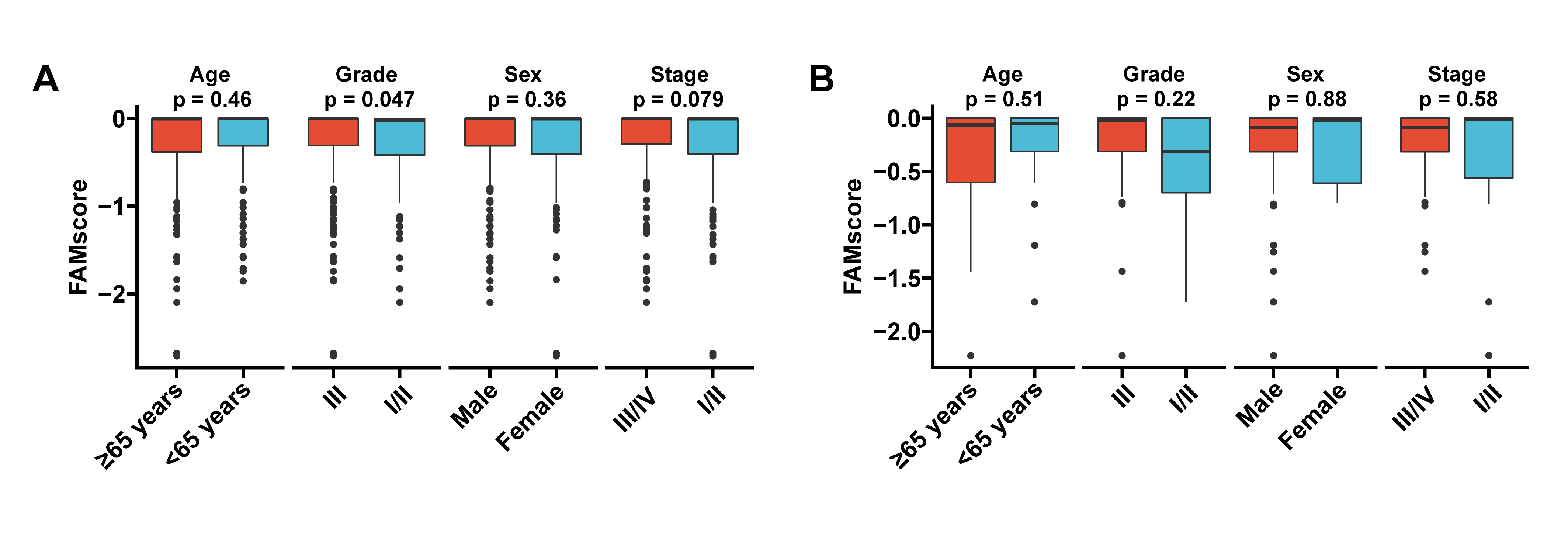

Supplement: Supplementary file 3 [file Image1.TIF]
